# Genetic and Cross Neutralization Analyses of Coxsackievirus A16 Circulating in Taiwan from 1998 to 2021 Suggest Dominant Genotype B1 can Serve as Vaccine Candidate

**DOI:** 10.3390/v14102306

**Published:** 2022-10-20

**Authors:** Dayna Cheng, Yo-Wei Chiu, Sheng-Wen Huang, Yun-Yin Lien, Chia-Lun Chang, Huey-Pin Tsai, Ya-Fang Wang, Jen-Ren Wang

**Affiliations:** 1 Institute of Basic Medical Sciences, College of Medicine, National Cheng Kung University, Tainan 70101, Taiwan; 2 National Institute of Infectious Diseases and Vaccinology, National Health Research Institutes, Tainan 70101, Taiwan; 3 National Mosquito-Borne Diseases Control Research Center, National Health Research Institutes, Tainan 70101, Taiwan; 4 Department of Pathology, National Cheng Kung University Hospital, College of Medicine, National Cheng Kung University, Tainan 70101, Taiwan; 5 Department of Medical Laboratory Science and Biotechnology, College of Medicine, National Cheng Kung University, Tainan 70101, Taiwan

**Keywords:** coxsackievirus A16, CVA16, epidemiology, phylogenetic analysis, recombination analysis

## Abstract

Coxsackievirus A16 (CVA16) is well known for causing hand-foot-and-mouth disease (HFMD) and outbreaks were frequently reported in Taiwan in the past twenty years. The epidemiology and genetic variations of CVA16 in Taiwan from 1998 to 2021 were analyzed in this study. CVA16 infections usually occurred in early summer and early winter, and showed increased incidence in 1998, 2000–2003, 2005, 2007–2008, and 2010 in Taiwan. Little or no CVA16 was detected from 2017 to 2021. CVA16 infection was prevalent in patients between 1 to 3 years old. A total of 69 isolates were sequenced. Phylogenetic analysis based on the VP1 region showed that CVA16 subgenotype B1 was dominantly isolated in Taiwan from 1998 to 2019, and B2 was identified only from isolates collected in 1999 and 2000. There was a high frequency of synonymous mutations in the amino acid sequences of the VP1 region among CVA16 isolates, with the exception of position 145 which showed positive selection. The recombination analysis of the whole genome of CVA16 isolates indicated that the 5′-untranslated region and the non-structural protein region of CVA16 subgenotype B1 were recombined with Coxsackievirus A4 (CVA4) and enterovirus A71 (EVA71) genotype A, respectively. The recombination pattern of subgenotype B2 was similar to B1, however, the 3D region was similar to EVA71 genotype B. Cross-neutralization among CVA16 showed that mouse antisera from various subgenotypes viruses can cross-neutralize different genotype with high neutralizing antibody titers. These results suggest that the dominant CVA16 genotype B1 can serve as a vaccine candidate for CVA16.

## 1. Introduction

Coxsackievirus A16 (CVA16) belongs to the species *Enterovirus A* (EV-A) of the *Picornaviridae* family. EV-A also includes CVA2 to CVA8, CVA10, CVA12, CVA14, enterovirus 71 (EVA71), EVA76, EVA89 to EVA92, EVA114, and EVA119 to EVA121. CVA16 and EVA71 are the two best known viruses which cause mild herpangina and hand-foot-and-mouth disease (HFMD) in young children. Several HFMD outbreaks associated with co-circulation of EVA71 and CVA16 have been reported in China, Japan, Korea, Malaysia, Singapore, Vietnam, and Thailand [1,2,3,4,5,6,7]. Of notice, there are three fatal reports of CVA16 infection: one is of a child who had HFMD associated with myocarditis, the second is of an adult who had pneumonitis, and the third is of a child who had HFMD associated with acute pancreatitis [8,9,10].

In Taiwan, CVA16 represented 15.5–35.8% of enterovirus infections from 1998 to 2005, except in 1999 (1.7%) and 2004 (1.7%) [11,12]. From 1998 to 2015, CVA16 as well as EVA71 were the most predominant enteroviruses associated with HFMD/herpangina cases [13,14,15,16]. Although CVA16 ranked as the top of both enterovirus infections and HFMD/herpangina cases in the past years, no information about the epidemiology and genetic analysis of CVA16 infection in Taiwan was reported.

CVA16 has a positive single-stranded RNA genome of 7410 nucleotides long. The viral genome contains 5′- and 3′-untranslated regions (UTR) and P1 to P3 regions. The P1 region encodes the VP1 to VP4 proteins which assemble the icosahedral capsid, and the capsid proteins are exposed on the virus surface, except for VP4. The P2 and P3 regions encode non-structural proteins including 2A to 2C and 3A to 3D, respectively [17]. VP1 contains the major antigenic sites and most of the serotype-specific neutralization determinants [18,19]. Molecular typing and genetic analysis based on VP1 sequence have been applied in a previous study, showing a high correlation between that and serotyping for enteroviruses [20]. Therefore, genetic analysis of CVA16 in the VP1 region is important to understand the strain variations among CVA16. Moreover, both humoral and cellular immunity induced by CVA16 can cross-react with EVA71, suggesting that common epitopes between EVA71 and CVA16 are probably located on the outer capsid proteins [21]. According to previous studies, the most extant phylogenetic analysis was constructed based on neighbor joining (NJ) [1,22]. CVA16 is divided into genotypes A, B and D based on the genetic diversity of the VP1 protein. Additionally, genotype B can be further divided into B1a, B1b, B1c, B2a, B2b, and B2c [23,24]. Clarification of the variations within the CVA16 VP1 protein can help to understand the antigenicity of CVA16.

Genomic recombination is a known phenomenon among enteroviruses as a mechanism to produce variants. Complete genomic sequences of all EV species suggested that intertypic or intratypic recombination occurred frequently in the non-structural regions to play a role in the evolution [25,26,27,28,29,30,31,32]. Recombination occurring in the regions encoding the non-structural proteins could potentially influence the replication, tissue tropism, and virulence of enteroviruses [33,34,35]. Two EVA71 isolates from Malaysia in 1997 and two isolates from China in 1998 and 2003 were shown to contain intertypic recombination between EVA71 and CVA16 in the non-structural region [36,37]. Intertypic recombination between EVA71 and CVA16 were also found in the 3C and 3D regions of 2008 Shenzhen and Fuyang EVA71 strains, respectively [23,38]. In addition, two CVA16 isolates were identified from Shenzhen in 2008 that contained intertypic recombination between CVA16 prototype G-10 and EVA71 genotype A at the 2A-2B junction [38]. Intertypic and intratypic recombination of circulating EVA71 in Taiwan have been reported and these studies suggested that recombination events could result in the emergence of viruses with altered potentials [39,40]. However, genomic recombination of circulating CVA16 in Taiwan has not been evaluated. These studies emphasized the importance of full-genome sequencing for the surveillance of CVA16 evolution.

In this study, we analyzed the annual and seasonal distributions of CVA16 isolated from 1998 to 2021 in Taiwan to present the epidemiology of CVA16 infection. The age and sex of CVA16 infected patients were also assessed. To evaluate the evolution of these Taiwan isolates, we performed phylogenetic analysis based on the VP1 coding region, analysis of complete genomic sequences, and recombination analysis between the Taiwan isolates and other EV-A. This long-term study providing information on the epidemiology and genetic evolution of CVA16 in Taiwan helps in the selection of a vaccine candidate for CVA16.

## 2. Materials and Methods

### 2.1. Viral Isolation and Identification

CVA16 isolates from the Virology Laboratory of National Cheng Kung University Hospital (NCKUH) from 1998 to 2021 were investigated. Clinical specimens from suspected EV-infected patients were inoculated into appropriate tissue cultures including human rhabodomyosarcoma (RD) (ATCC CCL-136) cells. The cytopathic effect (CPE) was examined for 10 days post-infection. When a 70% CPE of the cell monolayer was observed, the infected cells were harvested. Identification of CVA16 was performed by indirect immunofluorescent staining using Chemicon monoclonal antibodies including MAB979 (anti-Enterovirus 71 Antibody, cross-reacts with CVA16, clone 422-8D-4C-4D) and Millipore 3324 (Light Diagnostics Enterovirus 71 Monoclonal Antibody) [40].

### 2.2. Viral RNA Extraction

Based on the number of isolations at NCKUH every year, around five percent of the CVA16 isolates were selected each year for sequencing analyses. In addition, we performed complete genome analysis on half of those isolates. The CVA16 isolates were from a random sampling of patients with diverse clinical presentations, ranging from uncomplicated HFMD to CNS symptoms. CVA16 isolates were propagated in RD cells and the infected cells were harvested when a 75% CPE was seen. Viral genomic RNA was extracted from propagated viral culture fluid by using the Viral Nucleic Acid Extraction Kit (Geneaid, Taipei, Taiwan) according to manufacturer’s recommendation [40].

### 2.3. Sequence Analysis of CVA16

For cDNA synthesis of the VP1 coding region, a 12 µL reaction mixture of 10 µL of viral RNA, 1 µL of 10 mM dNTP mix, and 2 pmole 011 primer were heated at 65 °C for 5 min and chilled on ice immediately. Then, 4 µL of 5X First-Strand buffer (Invitrogen, Carlsbad, CA, USA), 2 µL of 0.1 M DTT, and 40 U of RNaseOUT (Invitrogen, Carlsbad, CA, USA) were added to the reaction mixture and incubated at 42 °C for 2 min. Finally, 200 U of SuperScript II Reverse Transcriptase (Invitrogen, Carlsbad, CA, USA) was added to a final volume of 20 µL. The reverse transcription (RT) reaction mixture was incubated at 42 °C for 50 min, heat-inactivated at 70 °C for 15 min, and then chilled on ice. For DNA amplification, the PCR primers 051 and 011 were used to amplify a 961 bp cDNA which includes the VP3, VP1, and 2A regions of the virus genome [20]. The PCR reaction contained 5 µL of cDNA, 1 µL of each 10 μM 051 and 011 primers, 5 µL of 10X PCR reaction buffer, 6.25 µL of 2.0 mM dNTP, 3 µL of 25 mM MgCl_2_, 1 U of DNA polymerase (KOD-Plus, TOYOBO, Osaka, Japan), and 27.8 µL of H_2_O. The PCR mixture was heated at 95 °C for 5 min and then 30 cycles consisting of 1 min at 95 °C, 1 min at 45 °C, and 1 min at 72 °C were carried out, followed by an additional 10 min incubation at 72 °C. PCR products were purified from agarose gel using PCR Product Purification Kit (Geneaid, Taipei, Taiwan). DNA sequencing was performed using 051 and 011 primers and the BigDye^®^ Terminator v3.1 Cycle Sequencing Kit (ABI, Foster City, CA, USA) on an ABI PRISM 3130XL Genetic Analyzer (ABI, Foster City, CA, USA).

The CVA16 full-length genome product was amplified as previously described [40]. Briefly, the viral genomic RNA was extracted and reverse transcribed with antisense primer RT-50 and SuperScript II Reverse Transcriptase (Invitrogen, Carlsbad, CA, USA). The cDNA was PCR-amplified with primers 4643-F (sense) and RT-50 by using DNA polymerase (KOD-Plus, TOYOBO, Osaka, Japan). PCR products were cloned using a TOPO XL PCR cloning kit (Invitrogen, Carlsbad, CA, USA) and sequenced. The specific primers used for RT-PCR and genome sequencing are indicated in Table S1 in the Supplemental Material. The sequences were assembled with the ContigExpress module of Vector NTI Advance 11 (Invitrogen, Carlsbad, CA, USA).

Multiple sequence alignments, including reference strains retrieved from GenBank (Table S2), were performed using the program Clustal W in Bioedit software version 7.1.3.0 and the evolutionary distances of nucleotide sequences were determined using the program DNADist within the software BioEdit, version 7.1.3.0 [41].

### 2.4. Site-Specific Selection Pressure and Estimation of Evolutionary Pathway

Single-likelihood ancestor counting (SLAC) and fixed effects likelihood (FEL) methods in the Datamonkey website “http://www.datamonkey.org (accessed on 21 September 2022)” were performed to examine non-synonymous (*dN*) and synonymous (*dS*) substitution rates for different genotypes and the selection signature in the CVA16 VP1 gene [42]. Selection pressure by the *dN/dS* ratio for each VP1 codon was measured, and *p*-values were also calculated at these residues. For all estimated values, a *p*-value of 0.1 was used as a statistical significance threshold to classify whether a site was negatively or positively selected. To determine the evolutionary pathway of VP1 protein, the ancestral states of CVA16 strains were reconstructed by using the probabilistic ASR method [43] via HyPhy online package [44]. Phylogenetic analysis was conducted by the Phylogenetic Analysis Using Parsimony (PAUP* 4.0) [45] and the results were visualized by Treeview software (version 1.6.6) [46]. Amino acid changes between lineages were determined and mapped along phylogenetic trees inferred for the VP1 gene.

### 2.5. Recombination Analysis

The procedure of recombination analysis was performed as previously described [40]. CVA16 recombination analysis used a transition/transversion rate of 10, and a 50% consensus to exclude the poorly conserved sites. Resulting alignments were analyzed using SimPlot version 3.5.1. BootScan analysis was run with a neighbor-joining tree algorithm and a Kimura distance model with 1000 pseudoreplicates.

### 2.6. Production of Reverse Genetics Viruses

Four reverse genetics (rg) viruses, rgH0041TW98 (B1a), rgM1136TW10 (B1a), rgN1771TW01 (B1b), and rg N1370TW00 (B2) were produced and sequence confirmed. Ten micrograms of DNA were linearized by using enzyme MluI and purified by phenol/chloroform (ratio 1:1). After linearization and DNA purification, the DNA was transferred to RNA by RiboMax large-scale RNA production system (Promega, WI, USA). The in vitro transcription reaction was terminated by adding 10 U RQ1 RNase and incubated for 30 min at 37 °C. The RNA was purified by phenol/chloroform extraction. RD cells were seeded (5 × 10^5^ cells/well) into 6-well plates for the following transfection step. Transfection of 2 µg of RNA into RD cell was performed by using TransMessenger transfection reagent (Qiagen, Taipei, Taiwan). The reverse genetics viruses were harvested when the cytopathic effect (CPE) was observed in more than 75% of the cells, as previously described. Reverse genetics viruses were used instead of native viruses for the immunization of mice to obtain anti-CVA16 serum, since reverse genetics viruses were more stable than native viruses which may contain a mixed quasispecies population.

### 2.7. TCID_50_ Assay 

The TCID_50_ is defined as the viral titer which causes 50% of cytopathic effect (CPE) to the cultured cells. To determine the virus TCID_50_ titer, we cultured 2 × 10^5^ RD cells/well into 96-well plates. Then, RD cells were infected with 100 μL of 10^−0.5^ serially diluted rg-viruses and incubated at 37 °C. Quadruplicate tests were examined for each viral titer. After five days of incubation, the TCID_50_ titer was calculated by the Reed-Muench method.

### 2.8. Preparation of Mouse Antisera

The reverse genetics viruses were cultured in RD cells. At least 500 mL of viral culture was collected and inactivated with UV radiation for 30 min, then centrifuged at 8000× *g* to remove the cell debris. The viral supernatant was mixed with 25 mL of 10% Nonidet P-40 (NP-40) and 500 mL of 16% polyethylene glycol (PEG) with 1M NaCl, then incubated overnight at 4 °C. The mixture was centrifuged for 10 min at 8000× *g* to collect the pellet. The protein pellet was resuspended in 50 mL of phosphate-buffered saline (PBS) and concentrated by Millipore centrifugal filters at 2500× *g* centrifugation, with the final volume being 2 mL. The collected viruses were quantified by Bradford protein assay and confirmed by Western blotting. One milligram protein of rg-viruses was used to immunize BALB/c mice with the addition of Freund’s complete adjuvant (CFA, Sigma, St. Louis, MO, USA) for priming and Freund’s incomplete adjuvant (IFA, Sigma, St. Louis, MO, USA). At the end of the immunization period, mice were sacrificed and the cardiac chambers serum sample were collected [47,48].

### 2.9. Neutralization Test

The neutralization tests were performed using the mouse antisera against the various linages of CVA16 viruses to test the neutralizing antibody titer. Mouse antisera were inactivated in a water bath for 30 min at 56 °C, and then two-fold serially diluted with viral medium. The diluted antiserum was incubated with 100 TCID_50_ virus for 2 h at 37 °C. Finally, 100 μL of antiserum-virus mixture was transferred to seeded RD cells and incubated at 37 °C for 5 days. The CPE observation was recorded daily and each titer was performed in duplicates [40].

### 2.10. Accession Numbers of the Nucleotide Sequences

All nucleotide sequences of CVA16 strains determined in this study were deposited to GenBank. The accession numbers are provided in Table S5.

## 3. Results

### 3.1. Epidemiology of CVA16 Infection in Southern Taiwan from 1998 to 2021

**Figure 1:**
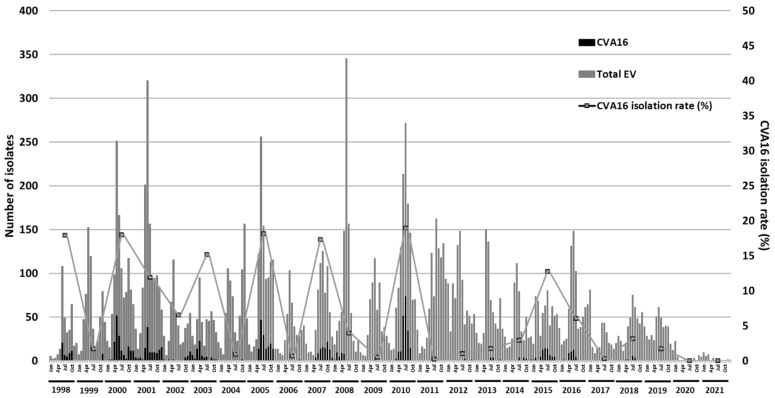
Monthly and yearly distribution of total enterovirus and CVA16 isolates in Southern Taiwan during 1998 to 2021. Numbers of total enterovirus isolates (grey column), CVA16 isolates (black column), and the CVA16 isolation rate (line graph) expressed.

A total of 1,156 CVA16 were identified among 14,650 enteroviruses isolated from the Clinical Virology Laboratory of National Cheng Kung University Hospital from 1998 to 2021. The annual distribution of total enterovirus infections and CVA16 infections from 1998 to 2021 are shown in Figure 1. Since the COVID-19 outbreak in 2019, CVA16 remained in low circulation. Of the annually isolated enterovirus infections, the proportion of CVA16 strains identified was 17.8% in 1998, 18.0% in 2000, 11.9% in 2001, 15.2% in 2003, 25.7% in 2005, 17.3% in 2007, 19.3% in 2010, 12.7% in 2015, 6% in 2016, and 3.2 % in 2018. The results showed that epidemic outbreaks of CVA16 occurred in Taiwan every 2–3 years with five peaks in 2000, 2001, 2005, 2007, and 2010. Little to no CVA16 activity was seen from 2017 to 2021.

To clarify the seasonal distribution of CVA16 infection throughout the observation period, monthly distribution of CVA16 infections from 1998 to 2021 are also shown in Figure 1. While no CVA16 infections were reported in 2020–2021, two peaks of CVA16 isolations were seen in the years 1998, 2000, 2001, and 2005 with one peak in early summer (May to June) and the other in late fall to early winter (October to December). One big peak pattern of CVA16 infections was observed in July of 2010. However, different patterns were seen in the 2002–2003 and the 2007–2008 outbreaks. In conclusion, CVA16 infections were distributed in the early summer and early winter with different seasonality patterns during the past two decades in Taiwan.

**Figure 2:**
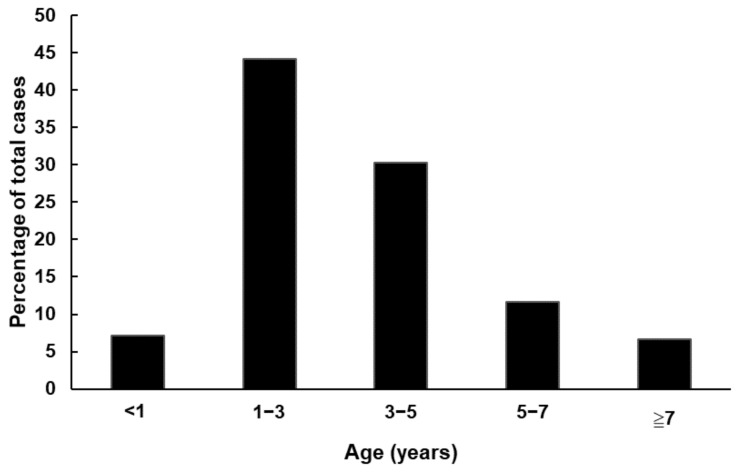
Age distribution of the patients infected with CVA16. The percentage of CVA16 infected cases in each group to total CVA16 cases was calculated and shown as column. The incidences of each group were listed as follows: 7.2% under 1 year old, 44.2% between 1 and 3 years old, 30.3% between 3 and 5 years old, 11.7% between 5 and 7 years old, and 6.6% more than 7 years old.

The age of CVA16 infected patients ranged from 1 month to 35 years old (age known for 964 patients) was identified. Approximately 44.2% of CVA16 infected cases were between 1 to 3 years old (Figure 2). However, the age distribution of patients during the different peaks of CVA16 infection throughout the years may differ. The case distribution according to gender showed that majority of the patients were males, with a sex ratio of 1.4 (463 males/341 females).

### 3.2. Phylogenetic Analysis of the VP1 Coding Region

**Figure 3:**
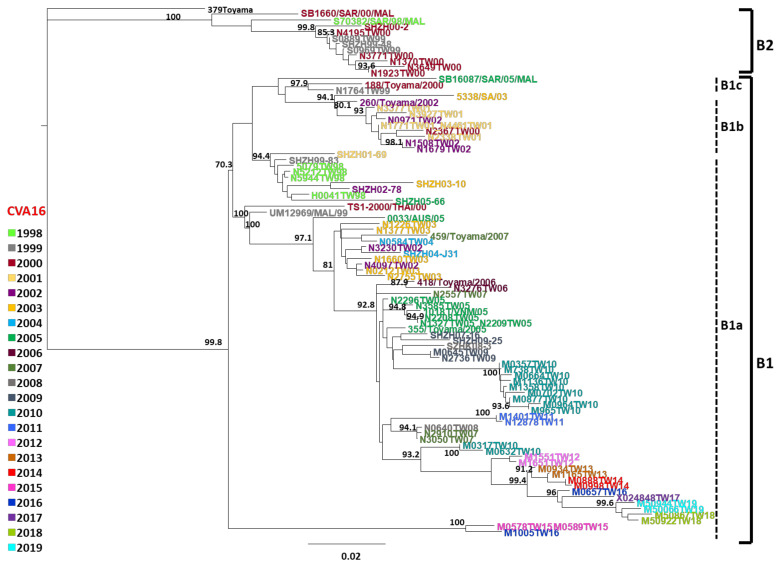
Phylogenetic analysis of CVA16 isolates from Taiwan and other countries based on the VP1 region (nt 2446-3325, 834 bp). A phylogenetic tree was estimated by the GTR model of the PAUP 4.0 program. Statistical robustness of 1,000 data sets were analyzed and statistical estimation of the significance of branch lengths was also determined by the maximum-likelihood method. Bootstrap values (percent of 1000 pseudoreplicate data sets) of over 75% supporting each cluster are shown at the nodes. CVA16 379/Toyama/1984 was included as an outgroup. Toyama: Japan; MAL: Malaysia; SZ and SHZH: Shenzhen, China; TW: Taiwan; SA: Saudi Arabia; AUS: Australia; THAI: Thailand; VNM: Vietnam.

Previous study indicated that CVA16 prototype G10 was quite different from other strains and was classified as genotype A. The nucleotide distances among the other CVA16 strains were less than 15%. These strains were designated as genotype B and were further divided into two groups: subgenotype B1 and B2 [23,49]. To examine the genotype of CVA16 circulating in Taiwan, a total of 69 CVA16 isolates were selected randomly from 1998 to 2019, as well as 25 CVA16 strains from other countries, to assess VP1 sequences using phylogenetic analysis and the pairwise comparison of the nucleotide sequences (Figure 3). The results indicated that distances of nucleotide sequences between subgenotype B1 and B2 were 7.7–15.6%. The cluster of subgenotype B1 composed of a Saudi Arabian strain from 2003, an Australian strain from 2005, a Thai strain from 2000, Malaysian strains from 1999 to 2005, Japanese strains from 1998 to 2007, Chinese strains from 1999 to 2009, and Taiwanese strains from 1998 to 2019 (Figure 3). The distances of nucleotide sequences within subgenotype B1 were 0.0–14.7%. The cluster of subgenotype B2 included the Malaysian strains from 1998 and 2000, Chinese strains from 1999 and 2000, Japanese strains from 1984, and Taiwanese strains from 1999 and 2000 (Figure 3). The distances of nucleotide sequences within subgenotype B2 were 0.2–9.8%.

The evolutionary relationship of the VP1 region between the CVA16 strains from Taiwan and from other countries was observed in the phylogenetic tree (Figure 3). It demonstrates that the CVA16 genotype B2 from Taiwan is very closely related to the Chinese strains from 1999 and 2000 (bootstrap value of 99.8%). Furthermore, three lineages of the CVA16 genotype B1 from Taiwan are divided by isolation year. The first lineage B1a consists of Taiwanese strains from 1998 which are clustered with Chinese strains from 1999 to 2005 (bootstrap value of 94.4%), and the recent Taiwanese strains from 2002 to 2019 which are much similar to a Thai strain from 2000, a Malaysian strain from 1999, Australian strains from 2005, Japanese strains from 2005 to 2007, and Chinese strains from 2005 to 2009 (bootstrap value of >97%). The second lineage B1b consists of Taiwanese strains from 1999 to 2002 which are grouped with the Saudi Arabian strain from 2003 and the Japanese strain from 2002 with a bootstrap value of 94.1%. Moreover, the Taiwanese strains from 2002 in the B1b cluster were isolated before July 2002 and differed with strains isolated after August 2002 which were in the B1a cluster.

### 3.3. Inference of Natural Selection among CV16 Strains

To examine the difference in amino acid sequences in the VP1 region among the selected 69 CVA16 isolates in Taiwan from 1998 to 2019, VP1 nucleotide sequences were translated to amino acid sequences using BioEdit 7.1.3.0. The distances of the nucleotide and amino acid sequences among these 69 CVA16 strains were 0.0–15.6% and 0.0–2.9%, respectively. There are only a few sporadic amino acid substitutions among these CVA16 strains in the capsid protein region, except for the greatest variation being at residue 145 of the VP1 protein. Glutamate (E), valine (V), glutamine (Q), glycine (G), and alanine (A) at site 145 were observed in 45, 17, 3, 2, and 1 CVA16 strains examined, respectively (Table 1). The residue 183 of the VP3 protein observed the alanine (A) to threonine (T) substitution between genotype B1 and B2. To determine the association between the nucleotide and amino acid sequence mutations in the VP1 protein coding regions, the mean *dN/dS* ratio of the 69 CVA16 sequences from this study was calculated using the SLAC analysis method. The result showed that the mean *dN/dS* ratio was 0.0484, indicating that most of the nucleotide substitutions were synonymous. To further identify the mutations involved in CVA16 VP1 evolution, SLAC and FEL analyses were performed to examine the *dN/dS* ratios of the individual sites in the VP1 protein coding region. Based on the SLAC analysis, 82 codons were identified as negatively selected sites, while 137 codons were characterized as being under a negative-selection force in FEL, respectively (Table S4). In conclusion, except for VP1-145, the comprehensive assessment of VP1 natural selection from the two methods identified no evidence of positive selection.

### 3.4. Whole Genome Analysis of CVA16 Isolated from Southern Taiwan from 1998–2016

Capsid proteins of enteroviruses are the major antigenic determinants and changes in the capsid proteins contribute to immune evasion. To determine whether the amino acid change contributes to the outbreaks, the deduced amino acid sequences of 32 selected viruses in Taiwan were aligned and compared based on the sequences of their polyprotein coding regions. The sequence comparisons of the whole capsid protein coding region showed two, two, and six amino acid variations in the VP2, VP3, and VP1 regions, respectively (Table 1). VP1 variations include N17S and V/E/Q/G/A 145, VP2 variations include V217I and A226T, and VP3 variations include P33S and A183T. There were other amino acid substitutions seen in VP1 including N17S, T164K, N218D, V251I, and T289I from 2012 to 2019. 

Alignment of polyprotein amino acid sequences revealed that the differences between subgenotypes B1 and B2 were mainly in the non-structural protein coding region. All 42 amino acid sequences in the non-structural protein coding region unique to subgenotype B2 were listed in Table S3, including 4, 7, 8, 1, 3, and 19 unique amino acids in the 2A, 2B, 2C, 3A, 3C, and 3D regions, respectively (Table S3). 

**Figure 4:**
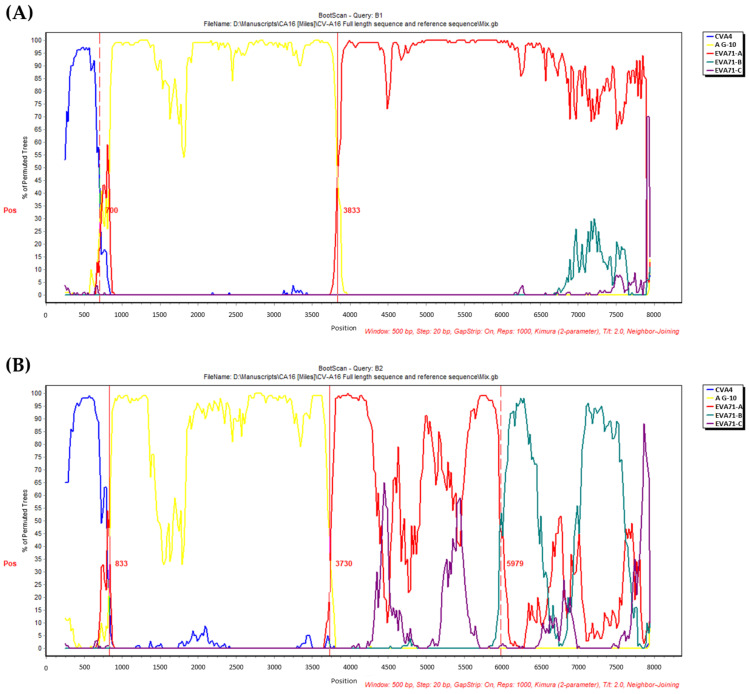
Bootscan analysis of CVA16 genotype B1 (**A**) and genotype B2 (**B**) compared with EV−A (CVA4/High Point, EVA71−A/BrCr, EVA71−B, EVA71−C, and CVA16/G10). The CVA16 genetic map is shown in the top panel. This analysis was calculated by SimPlot version 3.5.1 using the neighbor-joining tree algorithm (Kimura distance model) in a sliding window of 500 bp with a 20 bp step. Vertical lines and the numbers indicate the nucleotide position of the recombination crossover points.

To investigate the genome recombination of CVA16 isolated from Taiwan from 1998 to 2019, the complete genome sequences of CVA16 from southern Taiwan were aligned with other EV-A and six CVA16 strains isolated from China. Similarity plot and bootscan analysis were performed by using 50% consensus sequences of individual CVA16 isolated from 1998 to 2016 against other EV-A. Both analyses showed that all CVA16 strains clustering in the same subgenotype presented the same recombination phenomenon. Bootscan analysis revealed that there were two recombination crossover points with a high χ^2^ value within the genome of subgenotype B1 isolates. The first crossover point occurred in the 5′-UTR and the second was located in the 2A protein coding region, which supported the possibility of recombination events occurring in the 5′-UTR and the non-structural protein region. The 5′-UTR of CVA16 subgenotype B1 was more similar to Coxsackievirus A4. The non-structural protein region was similar to EVA71 genotype A BrCr strain, furthermore, the structural protein region was similar to CVA16 genotype A G10 strain supported by the high bootstrap value (Figure 4A). The pattern from the bootscan analysis performed with strains of subgenotype B2 was similar to B1 (Figure 4B). In addition, a third crossover point appeared in the 3C protein coding region and the 3D region was found to be similar to EVA71 genotype B with a high bootstrap value (Figure 4B). These results suggest that the 5′-UTR and 2A to 3C protein coding region of CVA16 subgenotype B1 and B2 were possibly recombined with CVA4 and EVA71 genotype A, respectively. The 3D region of CVA16 subgenotype B2 was possibly recombined with EVA71 genotype B which is different from subgenotype B1 with EVA71 genotype A. These results are consistent with the comparison of amino acid sequences which indicated that the non-structural protein regions of subgenotype B2 isolates were different from subgenotype B1.

### 3.5. Neutralizing Antibody Titers of rgCVA16-Induced Antisera against Various CVA16 and EVA71 Strains

For the neutralizing antibody titers test, we tested mouse antisera immunized with three different subgenotypes against twelve CVA16 isolates. The B1a rgCVA16 (H0041TW98) and B1a (M1136TW10) antisera showed 256 to 8192 neutralizing antibody titers against B1a, B1b, and B2 isolates. The B1b (N1771TW01) and B2 (N1370TW00) antisera showed much higher neutralizing antibody titer compared to B1a antiserum, ranging from 4096 to 131,072 neutralizing antibody titers. The B1a and B1b antisera showed <4 neutralizing antibody titer against EVA71 C2 and C4 isolates. The B2 antiserum showed 8192 neutralizing antibody titers against EVA71 C2 isolate and 16,384 neutralizing antibody titers against EVA71 C4 isolate (Table 2). These results showed that each subgenotype antiserum can cross-neutralize various genotypes. 

## 4. Discussion

In this study, a long-term surveillance of CVA16 infections in Taiwan from 1998 to 2021 was performed to demonstrate the epidemiology of several CVA16 outbreaks for more than twenty years. The incidence of CVA16 infections usually increased in early summer and early winter and the prevalent age of infected patients was between 1 to 3 years old, with a male to female ratio of 1.4. Phylogenetic analysis of the CVA16 isolates of CVA16 in Taiwan showed that there were two subgenotypes, B1 dominant and B2 minor, circulating in Taiwan. The genetic analysis showed that variation between the subgenotypes were mainly in the non-structural protein region and the lineage diversification were mainly attributed to synonymous substitutions in VP1 gene. Moreover, most amino acid sites of the VP1 evolved neutrally, with the exception of position 145 which showed positive selection. The CVA16 subgenotype B1 in Taiwan was shown to be recombined with CVA4 and EVA71 genotype A in the 5′−UTR and non-structural protein region, respectively, while subgenotype B2 was recombined with EVA71 genotype B in the non-structural protein region. Our recombination results were consistent with other previous reports [50,51,52]. In subgenotype B2, we saw the additional EVA71 genotype B recombination pattern in the nonstructural protein 3D (P3 region). Zhao et al. also demonstrated similar recombination pattern in the 3D protein [50]. Compared to our data, we showed the additional recombination pattern from 6000 bp to 6700 bp with EVA71 genotype B. These data revealed that CVA16 may recombine with other viruses due to co-circulation with other *Enterovirus A* viruses.

VP1 evolved under a highly conservative selection pressure in Coxsackievirus B5 (CVB5), despite the intergenotypic shifts associated closely with the immune selection against alternative genogroups [53]. Our study revealed a similar natural selection pattern of CVA16 VP1 region as CVB5; however, the CVA16 epidemic in Taiwan did not display codon replacement for the early genotype shift and later dominance of subgenotype B1 as that in CVB5. Concluding from the various phylodynamic patterns of epidemic pathogens, the extent of average immune selection force in hosts and pathogen adaptation capability would represent the strength of selection force shaping compositions and variation of pathogen diversity [54]. CVA16 outbreaks occurred almost every two years in Taiwan and Malaysia, and every 3-4 years in Japan [2,55,56]. The prevalent age of CVA16 infected patients might correlate to the epidemic interval, due to the dominant age of CVA16 infected children being less than three years old in Malaysia and Taiwan [56]. The evolution of CVA16 in Taiwan might suffer from high immune pressure that would limit the accumulation of amino acid changes and favor the escaped mutants in response to herd immunity, despite the boosting population size and genetic diversity inflated by fast substitution rate and high transmission rate [15].

Non-poliovirus enteroviruses (NPEV) were isolated throughout the year in tropical climates but circulated in the summer and fall in temperate climates [57,58]. Tseng et al. demonstrated that CVA16 detection generally rose between late spring and early summer in Taiwan from 2000 to 2005, but high CVA16 activities occurred in winter of 2002–2003 and in the fall of 2005 [11]. However, the monthly distribution of CVA16 infection also displayed a diverse pattern with infections in Taiwan increasing in early summer and early winter from 1998 to 2005. Factors that have been reported to facilitate enterovirus transmission include the high degree of humidity associated with elevated temperatures, and the rainfall rate leading to the contamination of drinking water [59,60]. To prevent CVA16 infections efficiently, the factors that affect the CVA16 epidemic should be further investigated.

Phylogenetic analysis of the VP1 region of CVA16 strains in Taiwan from 1998 to 2019 indicated that Taiwan strains were divided into two groups designated as genotype B1 from 1998 to 2019 and B2 which co-circulated with B1 from 1999 to 2000, respectively. Molecular typing of CVA16 infections has been reported in the Asia-Pacific region, including China, Japan, Malaysia, Thailand, and Taiwan [55,61,62,63,64,65,66]. Despite temporal shift to genotype B2, the dominant genotype was B1 among countries, irrespective of geographic distance (Table 3). Additionally, all three lineages of subgenotype B1 from Taiwan are closely related to CVA16 strains from different countries (Figure 3). Accordingly, we suggest that similar temporary dynamics found in Asia among CVA16 genotypes, irrespective of geography and ethnology, might largely result from the traffic convenience to homogenize the epidemic signature on those susceptible populations.

The results from SLAC and FEL analyses demonstrated that CVA16 in Taiwan from 1998 to 2019 had a high frequency of synonymous mutation. It indicated that the residues of the VP1 protein were quite stable and only a high variation of the residue was observed at position 145. The VP1-145 was shown to be a positive selection site and the same finding was also observed in the EVA71 studies [40,67]. The amino acid residue at position 145 has been proven to be located in the surface-exposed DE loop of the EVA71 VP1 protein which is one of the major antigenic sites [18,68]. The substitution (G to E) at residue 145 in the VP1 protein for mouse-adapted EVA71 plays a role in viral binding, uncoating efficiency, and RNA accumulation in infected cells [69,70,71]. Additionally, VP1-145 is the key residue affecting EVA71 binding to PSGL-1 and play a role in heparan binding [72]. Lal et al. reported that the VP1 region spanning amino acids 66-297 is an interaction domain for EVA71 VP1 self-association [73]. VP1-145Q may result in the strengthening of the VP1 self-association, which changes the VP1 structure and then efficiently affects the viral binding and uncoating to the infected cells in CNS. This observation correlates with previous studies which showed that amino acids G/Q/A at position 145 of VP1 are associated with virulent phenotype by comparing the genomic sequences of EVA71 derived from severe cases and mild cases [74,75].

The sequential comparisons of the whole capsid protein coding region (Table 1) suggest that the CVA16 epidemics was possibly caused by accumulated amino acid changes in capsid proteins. However, we found contrasting pattern of nonsynonymous substitutions in VP1 phylogenetic tree since most variations were clustered exclusively near the phylogenetic terminus of subgenotype B1, suggesting that the intragenotype changes might not correlate with antigenic shift. In addition to the scattered distribution of amino acid variations found along the VP1 region, the reconstructed ancestral amino acid composition in internal nodes showed diversification of CVA16 [24,76]. The major antigenic epitopes of CVA16 might have existed on VP2 or VP3 region, instead of VP1 [77,78]. In addition to the substitution A183T in VP3, the alternative recombination with EVA71 genotype B occurring in the non-structural protein region (Figure 4) possibly contributed to the outbreak of the CVA16 subgenotype B2 in 2000, suggesting that the outbreak may be due to the alteration of the virulence. 

The recombination between EVA71 and CVA16 was also reported in the studies of other countries such as Japan, Malaysia, Singapore, and China [32,36,37,38,39,40,79]. Chan and AbuBakar demonstrated that intertypic recombination occurred between EVA71 and CVA16 [36,61]. Intertypic recombination was also shown in Japan and Taiwan in EVA71 genotype C2 [40,79,80]. In China, Zhang et al. demonstrated that intertypic recombination of subgenotype C4 (including C4a and C4b) EVA71 with CVA16, CVA14, and CVA4 [81]. Yip et al. also showed double recombination between EVA71 genotype B, C and a CVA16 G-10 strain [38]. Consistent with these studies, the recombination break point occurred in the P2 and P3 region between CVA16 and EVA71 genome [32,80,82].

In China, previous studies showed that the anti-EVA71 antibody can cross-neutralize CVA16 [83,84]. The neutralization titer from the infected children anti-EVA71 IgM also showed much lower titer to anti-CVA16 [84]. Yang et al. tested the combined vaccine of EVA71 and CVA16, and each vaccine individually against the virus infection in monkey. The results showed that only the CVA16 vaccine can provide a better protection over the EVA71 or combined vaccine [85]. Therefore, a suitable CVA16 vaccine candidate is an unmet need. In this study, various anti-CVA16 antibody can cross-neutralize different subgenotypes of CVA16. Interestingly, only anti-CVA16 subgenotype B2 antibody can cross-neutralize EVA71. 

The identification of the amino acid substitutions in capsid proteins arouses the possibility of the viral antigenic changes contributing to the re-emergent outbreaks. However, the antigenic sites of CVA16 are still unknown which urges the determination and comparison of antigenicity between isolates and genotypes. BootScan analysis and the comparison of whole viral genome sequences revealed the differences in the non-structural protein region between subgenotype B1 and B2. These residues are possible determinants that may affect replication, tissue tropism, and virulence of the virus. Reverse genetics and corresponding functional assays in this study will be helpful to identify the virulence determinants of CVA16. In conclusion, recognition of the CVA16 infection in epidemiology and genetic variation will provide more information in the treatment of the disease, the relationship between EVA71 and CVA16, and help with the future development of a CVA16 vaccine.

## Figures and Tables

**Table 1:** Amino acid sequence comparison of capsid protein of CVA16 in Taiwan.

Virus Genotype and Isolates	VP1 ^a^						VP2		VP3	
	17 ^b^	145	164	218	251	289	217	226	33	183
**Subgenotype B1**										
H0041TW98	N	V	T	N	V	T	V	A	P	A
5079TW98 ^c^	N	E	T	N	V	T	V	A	P	A
N1764TW99	N	E	T	N	V	T	I	A	P	A
N2367TW00	N	V	T	N	V	T	I	T	P	A
N1771TW01 ^d^	N	E	T	N	V	T	I	A	P	A
N1508TW02 ^e^	N	E	T	N	V	T	I	A	P	A
N1679TW02 ^f^	N	V	T	N	V	T	I	A	P	A
N1660TW03 ^g^	N	E	T	N	V	T	I	A	P	A
N0584TW04	N	V	T	N	V	T	I	A	P	A
N2208TW05	N	V	T	N	V	T	I	T	P	A
N3276TW06	N	V	T	N	V	T	I	T	S	A
N2910TW07 ^h^	N	E	T	D	V	T	I	T	P	A
N0640TW08	N	V	T	D	V	T	I	T	P	A
M0645TW09 ^i^	N	E	T	N	V	T	V	T	P	A
M0317TW10	N	E	T	N	V	T	I	T	P	A
M0664TW10 ^j^	N	E	T	N	V	T	I	T	S	A
M0964TW10	N	L	T	N	V	T	I	T	S	A
M1401TW11	N	E	T	N	V	T	V	A	P	A
N12878TW11	N	V	T	N	V	T	V	A	P	A
M1551TW12	N	Q	K	N	I	I	V	T	P	A
M1651TW12	N	V	K	N	I	I	V	T	P	A
M0934TW13	N	V	K	N	I	T	V	T	P	A
M1165TW13	N	E	K	N	I	T	V	T	P	A
M0888TW14	S	E	K	N	I	T	V	T	P	A
M0998TW14	S	E	K	N	I	T	V	T	P	A
M0578TW15	N	E	T	N	I	T	I	T	P	A
M0589TW15	N	E	T	N	V	T	I	T	P	A
M1005TW16	N	E	T	N	V	T	I	T	P	A
M0657TW16	N	Q	K	N	I	T	V	T	P	A
X024848TW17	N	A	K	N	I	T	V	T	L	A
M50922TW18	N	E	K	N	I	T	V	T	P	A
M50867TW18	N	E	K	N	I	T	V	T	P	A
M50944TW19	N	G	K	N	I	T	V	T	P	A
M50066TW19	N	E	K	N	I	T	V	T	P	A
**Subgenotype B2**										
S0969TW99	N	V	T	N	V	T	V	A	P	T
N1370TW00 ^k^	N	E	T	N	V	T	V	A	P	T
N3649TW00	N	Q	T	N	V	T	V	A	P	T

^a^ CVA16 viral protein coding region. ^b^ The numbers represents the amino acid position in the viral protein. ^c^ The same sequence of VP1 was also identified in N5212TW98, and N5944TW98. ^d^ The same sequence of VP1 was also identified in N3377TW01, N3927TW01, and N4461TW01. ^e^ The same sequence of VP1 was also identified in N3230TW02 and N4097TW02. ^f^ The same sequence of VP1 was also identified in N0971TW02, N1226TW03, and N1377TW03. ^g^ The same sequence of VP1 was also identified in N0212TW03, and N2755TW03. ^h^ The same sequence of VP1 was also identified in N3050TW07. ^i^ The same sequence was also identified in N2736TW09. ^j^ The same sequence was also identified in M0357TW10, M0632TW10, M0702TW10, M0738TW10, M0877TW10, M0965TW10, M1136TW10, and M1358TW10. ^k^ The same sequence of VP1 was also identified in S0889TW99, N1923TW00, and N3771TW00.

**Table 2:** Neutralizing antibody titers of rgCVA16 -induced antisera against various CVA16 and EVA71 strains.

Virus Tested	Neutralizing Antibody Titer of Antiserum			
	rgH0041TW98 (B1a)	rgM1136TW10 (B1a)	rgN1771TW01 (B1b)	rgN1370TW00 (B2)
N5212TW98 (B1a)	4096	4096	32,768	16,384
N5944TW98 (B1a)	512	1024	16,384	16,384
H0041TW98 (B1a)	4096	2048	131,072	32,768
M965TW10 (B1a)	4096	4096	32,768	16,384
M0664TW10 (B1a)	512	512	16,384	32,768
M738TW10 (B1a)	512	512	8192	32,768
N3377TW01 (B1b)	8192	4096	32,768	16,384
N3927TW01 (B1b)	512	512	8192	16,384
N1923TW00 (B2)	512	512	4096	8192
N3771TW00 (B2)	256	512	4096	65,536
N3649TW00 (B2)	1024	4096	65,536	16,384
N1370TW00 (B2)	512	2048	65,536	8192
EVA71 (C2)	<4	<4	<4	8192
EVA71 (C4)	<4	<4	<4	16,384

**Table 3:** The summary of CVA16 genotype based on VP1 region circulated in different countries from 1984 to 2019.

	1984–1996	1997	1998	1999	2000	2001	2002	2003	2004	2005	2006	2007	2008	2009	2010	2011	2012	2013	2014	2015	2016	2017	2018	2019
**Japan**	**A, B1, B2^a^**	B1, B2	B1, **B2**	B1	**B1**	B1	**B1**	B1	B1	B1	B1	B1	B1	B1	B1	B1								
**China**				B1, B2	B1, B2	B1	B1	B1	B1	B1	B1	B1	B1	B1	B1		B1	B1						
**Malaysia**		B1	B1, B2	B1	B1, B2	B1	B1	B1		B1	B1	B1												
**Vietnam**										B1														
**Thailand**					B1									B1	B1	B1								
**Australia**				B1																				
**Saudi Arabia**						B1		B1																
**Taiwan**			**B1**	B1, B2	B1, **B2**	**B1**	B1	**B1**	B1	**B1**	B1	**B1**	B1	B1	**B1**	B1	B1	B1	B1	B1	B1	B1	B1	B1

^a^ The letter in bold means the predominant genotype in the outbreak.

## Data Availability

The data that support the findings of this study are available within the article.

## Supplementary Materials

The following supporting information can be downloaded at: https://www.mdpi.com/article/10.3390/v14102306/s1, Table S1: The specific primers used for RT-PCR and genome sequencing. Table S2: The reference strains obtained from Genbank used in phylogenetic analysis and BootScan analysis. Table S3: Non-structural protein amino acid sequences specific for CVA16 in Taiwan. Table S4: Summary of the number of codons identified by single-likelihood ancestor counting (SLAC) and fixed effects likelihood (FEL) methods. Table S5: Total CVA16 isolation Accession Numbers.
