# Supplementary material for: Genetic and Cross Neutralization Analyses of Coxsackievirus A16 Circulating in Taiwan from 1998 to 2021 Suggest Dominant Genotype B1 can Serve as Vaccine Candidate
Source: Viruses. 2022 Oct 20;14(10):2306. doi: 10.3390/v14102306 (PMC9608817; doi:10.3390/v14102306)
Supplement: Supplementary file 1 [file viruses-14-02306-s001.zip › viruses-1904129-supplementary.pdf]

Table S1. The specific primers used for RT-PCR and genome sequencing.

Primer	Sequence
**CA16-679F**	GGATACRTCCCTCTYAATYWCAAG
**CA16-1312F**	GCTTTRCTRGTRGCYGTG
**CA16-2054F**	CATTRGARGTRACRTTYATG
**CA16-2092F**	GCYACAGGYAAGATGCTYATYG
**CA16-3201F**	GGATTAAACACGTCAGAGCATGG
**CA16-3384R**	CTACYCTRTARTTGCCBACATATATGGC
**CA16-3821F**	GGTATGGGTTTCACTGATGC
**CA16-4414F**	GCAGTTCAAGAGCAAACAC
**CA16-5272F**	GCAGTTGTGTCYCTTGTCTATG
**CA16-5294F**	GTTATTTACAAATTGTTCGCYGG
**CA16-5444R**	GCACCTGTCTAATGTTACGCC
**CA16-5922F**	CTTYGCCAGTGAACARGGAG
**CA16-6017F**	CCCAGTGYATTCYATGATG
**CA16-6722F**	GCTGTGTCACTAATAGAAGGG
**CA16-6804R**	CCTGAGGGCATTCCACCAAG
**CA16-7401R**	GGTTATAACAAATTTACCCCCACC
**4643F**	GTAGAATTCAGCTAATACGACTCACTATAGTTAAAACAGCCTGTGGGTTG
**4643R-T50**	CCTACGGCCGTTTTTTTTTT TTTTTTTTTTTTTTTTTTTTTTTTTTTTTTTTTTTTTTTGCTATTCTGGTTATAAC AATTTAC
**051**	TSAARYTGTGCAARGACAC
**011**	GCICCIGAYTGITGICCRAA

Table S2. The reference strains obtained from Genbank used in phylogenetic analysis and BootScan analysis

Isolate	Year of isolation	Country	Accession Number
S70382*	1998	Malaysia	AM292461
UM12969*	1999	Malaysia	AM292479
SB1660*	2000	Malaysia	AM292465
SB16087*	2005	Malaysia	AM292528
Shzh99-48*	1999	China	AY895116
Shzh99-83*	1999	China	AY821797
Shzh00-2*	2000	China	AY895127
Shzh01-69*	2001	China	AY895111
Shzh02-78*	2002	China	AY895108
Shzh03-10*	2003	China	AY895095
Shzh04-J31*	2004	China	AY821796
Shzh/05-66*	2005	China	HM776224
Shzh/07-16*	2007	China	HM776248
SZ/HK08-3*	2008	China	GQ279368
Shzh/09-2-5*	2009	China	HM776277
0033*	2005	Australia	AM292435
TS1-2000*	2000	Thailand	AM292477
5338*	2003	Saudi Arabia	AM292443
1018T*	2005	Vietnam	AM292441
379/Toyama*	1984	Japan	AB465367
188/Toyama*	2000	Japan	AB465372
260/Toyama*	2002	Japan	AB465389
355/Toyama*	2005	Japan	AB465401
418/Toyama*	2006	Japan	AB465402
459/Toyama*	2007	Japan	AB465404
CA16/G-10*†	1951	South Africa	U05876
CA4/High Point†	1948	USA	AY421762
EV71/BrCr (A)†	1970	USA	U22521
EV71/237-TW86 (B1)†	1986	Taiwan	FJ357380
EV71/MS/7423/87 (B2)†	1987	USA	U22522
EV71/SAR/SHA66 (B3)†	1997	Malaysia	AM396586
EV71/S0318-TW01 (B4)†	2001	Taiwan	FJ357376
EV71/M0380-TW08 (B5)†	2008	Taiwan	FJ357385
EV71/804/NO/03 (C1)†	2003	Norway	DQ452074
EV71/ N4643-TW98 (C2)†	1998	Taiwan	AF304458
EV71/06-KOR-00 (C3)†	2000	South Korea	DQ341355
EV71/GZ-08-02 (C4)†	2008	China	FJ360545
EV71/E2005125-TW (C5)†	2006	Taiwan	EF063152

* Sequences analyzed in phylogenetic analysis.
† Sequences analyzed in Bootscan analysis.
( ) The corresponding EV71 genotypes are described in the brackets

Table S3. Non-structural protein amino acid sequences specific for CV-A16 in Taiwan.

Virus genotype and isolates	2A[a]				2B							2C								3A
	82[b]	85	102	123	3	6	15	57	85	95	96	23	102	103	228	243	280	303	311	39
Subgenotype B1																				
H0041TW98[c]	I	E	V	I	S	I	V	V	A	I	V	S	Y	A	V	Y	R	I	I	E
N1764TW99	I	E	V	I	S	I	M	V	A	I	V	S	Y	A	V	Y	R	I	I	E
N2367TW00	I	E	V	I	S	I	M	V	A	I	V	S	Y	A	V	Y	R	I	I	E
N1771TW01[d]	I	E	V	I	S	I	M	V	A	I	V	S	Y	A	V	Y	R	I	I	E
N1660TW03	I	E	V	I	S	I	M	V	A	I	V	S	Y	A	V	Y	R	I	I	E
N0584TW04	I	E	V	I	S	I	M	V	A	I	V	S	Y	A	V	Y	R	I	I	E
N2208TW05[e]	I	E	V	I	S	I	M	V	A	I	V	S	Y	A	V	Y	R	I	I	E
N2910TW07	I	E	V	I	S	I	M	V	A	I	V	S	Y	A	V	Y	R	I	I	E
N0640TW08	I	E	V	I	S	I	M	V	A	I	V	S	Y	A	V	Y	R	I	I	E
M0645TW09[f]	I	E	V	I	S	I	M	V	A	I	V	S	Y	A	V	Y	R	I	I	E
M0317TW10	I	E	V	I	S	I	M	V	A	I	V	S	Y	A	V	Y	R	I	I	E
M0664TW10[g]	I	E	V	I	S	I	M	V	A	I	V	S	Y	A	V	Y	R	I	I	E
M1401TW11[h]	I	E	V	I	S	I	M	V	A	I	V	S	Y	A	V	Y	R	I	I	E
M1651TW12	I	E	V	I	S	I	M	V	A	I	V	S	Y	A	V	Y	R	I	I	E
M1165TW13	I	E	V	I	S	I	M	V	A	I	V	S	Y	A	V	Y	R	I	I	E
M0888TW14	I	E	V	I	S	I	M	V	A	I	V	S	Y	A	V	Y	R	I	I	E
M0589TW15	I	E	V	I	S	I	M	V	A	I	V	S	Y	A	V	Y	R	I	I	D
M0657TW16	I	E	V	I	S	I	M	V	A	I	V	S	Y	A	V	Y	R	I	I	E
Subgenotype B2																				
N1370TW00[i]	V	D	Q	L	T	V	I	I	S	M	A	N	F	T	I	F	K	V	V	D
N3649TW00	V	D	Q	L	T	V	I	I	S	M	A	N	F	T	I	F	K	V	V	D

a: CA16 viral protein coding region.
b: The numbers represents the amino acid position in the viral protein.
c: The same sequence was also identified in 5079TW98.
d: The same sequence was also identified in N1508TW02, and N1679TW02.
e: The same sequence was also identified in N3276TW06.
f: The same sequence was also identified in N2736TW09.
g: The same sequence was also identified in M0357TW10, M0632TW10, M0702TW10, M0877TW10, M0964TW10, and M1136TW10.
h: The same sequence was also identified in N12878TW11.
i: The same sequence was also identified in S0969TW99.

Table S3. Non-structural protein amino acid sequences specific for CV-A16 in Taiwan (continued).

Virus genotype and isolates	3C			3D																		
	93	95	177	37	66	68	75	89	91	106	125	134	138	139	168	197	251	255	279	342	396	428
Subgenotype B1																						
H0041TW98	T	T	G	N	T	H	T	D	N	Y	V	I	T	T	I	T	V	E	K	S	R	E
N1764TW99	T	T	G	N	T	H	T	D	N	Y	V	I	T	T	I	T	V	E	R	S	R	E
N2367TW00	T	T	G	N	T	H	T	D	N	Y	V	I	T	T	I	T	V	E	K	S	R	E
N1771TW01	T	T	G	N	T	H	T	D	N	H	V	I	T	T	I	T	V	E	K	S	R	E
N1660TW03	T	T	G	N	T	H	T	D	N	Y	V	I	T	A	I	T	V	E	K	S	R	E
N0584TW04	T	T	G	N	T	H	T	D	N	Y	I	T	T	T	I	T	V	E	K	S	R	E
N2208TW05	T	T	G	S	T	H	T	D	N	Y	V	I	T	T	I	T	V	E	K	S	R	E
N2910TW07	T	T	S	S	T	H	T	D	N	Y	I	I	T	T	I	T	V	E	K	S	R	E
N0640TW08	T	T	S	S	T	H	T	D	N	Y	V	I	T	T	I	T	V	E	K	S	R	E
M0645TW09	T	T	S	S	T	H	T	D	N	Y	V	I	T	T	I	T	V	E	K	S	R	E
M0317TW10	T	T	S	S	T	H	T	D	N	Y	V	V	T	T	I	T	M	E	K	S	R	E
M0664TW10	T	T	S	S	T	H	T	D	N	Y	V	T	T	T	I	T	V	E	K	S	R	E
M1401TW11	T	T	S	I	T	H	T	D	N	Y	V	I	T	T	I	T	L	E	K	S	R	E
M1651TW12	T	T	G	S	T	H	M	D	N	Y	I	V	T	T	I	T	V	E	K	S	R	E
M1165TW13	T	T	G	S	T	H	T	D	N	Y	I	V	T	T	I	T	V	E	K	S	R	E
M0888TW14	T	T	G	S	T	H	M	D	N	Y	I	V	T	T	I	T	V	E	K	S	R	E
M0589TW15	T	T	S	N	T	H	T	D	N	Y	V	I	T	T	I	T	V	E	K	S	K	E
M0657TW16	T	T	G	S	T	H	T	D	N	Y	I	V	T	T	I	T	V	E	K	S	R	E
Subgenotype B2																						
N1370TW00	D	S	S	D	V	Y	Q	S	D	N	I	T	V	S	V	V	M	D	R	L	K	D
N3649TW00	D	S	S	D	V	Y	Q	S	N	N	I	T	V	S	V	V	M	D	R	L	K	D

Table S4. Summary of the number of codons identified by Single-likelihood ancestor counting (SLAC) and fixed effects likelihood (FEL) methods.

**Number of sites**	**SLAC (*p*<0.1)**	**FEL (*p*<0.1)**
Positively selected	1	1
Negatively selected	82	137

Table S5. Total CV-A16 isolation Accession Numbers.

Isolate	Year of Isolation	Accession Number
N4195TW00	2000	OP562202 (VP1)
S0889TW99	1999	OP562203 (VP1)
N1923TW00	2000	OP562204 (VP1)
N3771TW00	2000	OP562205 (VP1)
N5212TW98	1998	OP562206 (VP1)
N5944TW98	1998	OP562207 (VP1)
N2338TW01	2001	OP562208 (VP1)
N3377TW01	2001	OP562209 (VP1)
N3927TW01	2001	OP562210 (VP1)
N4461TW01	2001	OP562211 (VP1)
N0971TW02	2002	OP562212 (VP1)
N3230TW02	2002	OP562213 (VP1)
N4097TW02	2002	OP562214 (VP1)
N0212TW03	2003	OP562215 (VP1)
N2755TW03	2003	OP562216 (VP1)
N1226TW03	2003	OP562217 (VP1)
N1377TW03	2003	OP562218 (VP1)
N2557TW07	2007	OP562219 (VP1)
N2296TW05	2005	OP562220 (VP1)
N3585TW05	2005	OP562221 (VP1)
N1327TW05	2005	OP562222 (VP1)
N2209TW05	2005	OP562223 (VP1)
M738TW10	2010	OP562224 (VP1)
M965TW10	2010	OP562225 (VP1)
M1358TW10	2010	OP562226 (VP1)
N3050TW07	2007	OP562227 (VP1)
M1551TW12	2012	OP562228 (VP1-VP4)
M0934TW13	2013	OP562229 (VP1-VP4)
M0998TW14	2014	OP562230 (VP1-VP4)
M0578TW15	2015	OP562231 (VP1-VP4)
M1005TW16	2016	OP562232 (VP1-VP4)
M50867TW18	2018	OP562233 (VP1-VP4)
M50922TW18	2018	OP562234 (VP1-VP4)
M50066TW19	2019	OP562235 (VP1-VP4)
X024848TW17	2017	OP562236 (VP1-VP4)
M50944TW19	2019	OP562237 (VP1-VP4)
5079TW98	1998	AF177911 (Full Length)
S0969TW99	1999	OP562188 (Full Length)

N1370TW00	2000	OP562189 (Full Length)
N3649TW00	2000	OP562190 (Full Length)
H0041TW98	1998	OP562170 (Full Length)
N1764TW99	1999	OP562171 (Full Length)
N2367TW00	2000	OP562172 (Full Length)
N1771TW01	2001	OP562173 (Full Length)
N1508TW02	2002	OP562174 (Full Length)
N1679TW02	2002	OP562175 (Full Length)
N1660TW03	2003	OP562176 (Full Length)
N0584TW04	2004	OP562177 (Full Length)
N3276TW06	2006	OP562179 (Full Length)
N2208TW05	2005	OP562178 (Full Length)
M0645TW09	2009	OP562182 (Full Length)
N2736TW09	2009	OP562191 (Full Length)
M0357TW10	2010	OP562193 (Full Length)
M0664TW10	2010	OP562184 (Full Length)
M0702TW10	2010	OP562194 (Full Length)
M0877TW10	2010	OP562195 (Full Length)
M0964TW10	2010	OP562185 (Full Length)
M1136TW10	2010	OP562196 (Full Length)
N2910TW07	2007	OP562180 (Full Length)
N0640TW08	2008	OP562181 (Full Length)
M1401TW11	2011	OP562186 (Full Length)
N12878TW11	2011	OP562187 (Full Length)
M0317TW10	2010	OP562183 (Full Length)
M0632TW10	2010	OP562192 (Full Length)
M1651TW12	2012	OP562197 (Full Length)
M1165TW13	2013	OP562198 (Full Length)
M0888TW14	2014	OP562199 (Full Length)
M0589TW15	2015	OP562200 (Full Length)
M0657TW16	2016	OP562201 (Full Length)
